# Fast and simple high-capacity quantum cryptography with error detection

**DOI:** 10.1038/srep46302

**Published:** 2017-04-13

**Authors:** Hong Lai, Ming-Xing Luo, Josef Pieprzyk, Jun Zhang, Lei Pan, Shudong Li, Mehmet A. Orgun

**Affiliations:** 1 School of Computer and Information Science and Centre for Research and Innovation in Software Engineering, Southwest University, Chongqing 400715, China; 2 School of Information Science and Technology, Southwest Jiaotong University, Chengdu 610031, China; 3 School of EE&CS, Queensland University of Technology, Brisbane, Australia and Institute of Computer Science, Polish Academy of Sciences, Warsaw, Poland; 4 School of Information Technology, Deakin University, Geelong, VIC, 3220, Australia; 5 College of Mathematics and Information Science, Shandong Institute of Business and Technology, Yantai, Shandong 264005, China; 6 School of Computer Science,National University of Defense Technology, 410073 Changsha, China; 7 Department of Computing, Macquarie University, Sydney, NSW 2109, Australia; 8 Faculty of Information Technology, Macau University of Science and Technology, Avenida Wai Long, Taipa, 999078, Macau

## Abstract

Quantum cryptography is commonly used to generate fresh secure keys with quantum signal transmission for instant use between two parties. However, research shows that the relatively low key generation rate hinders its practical use where a symmetric cryptography component consumes the shared key. That is, the security of the symmetric cryptography demands frequent rate of key updates, which leads to a higher consumption of the internal one-time-pad communication bandwidth, since it requires the length of the key to be as long as that of the secret. In order to alleviate these issues, we develop a matrix algorithm for fast and simple high-capacity quantum cryptography. Our scheme can achieve secure private communication with fresh keys generated from Fibonacci- and Lucas- valued orbital angular momentum (OAM) states for the seed to construct recursive Fibonacci and Lucas matrices. Moreover, the proposed matrix algorithm for quantum cryptography can ultimately be simplified to matrix multiplication, which is implemented and optimized in modern computers. Most importantly, considerably information capacity can be improved effectively and efficiently by the recursive property of Fibonacci and Lucas matrices, thereby avoiding the restriction of physical conditions, such as the communication bandwidth.

Quantum cryptography provides a feasible solution to the key generation and key management issues for one-time pad (OTP) encryption. Recall that for the classical implementation of OTP, a cryptographic key needs to be generated and distributed to the communicating parties via a secure channel and well ahead of the use of OTP. This constraint is no longer valid in the quantum setting. The randomness necessary to create and distribute a cryptographic key is readily obtained by the parties from observations of quantum signals exchanged between the parties. The quantum key distribution (QKD) provides a straightforward implementation of OTP which preserves its unconditional security^1^,^2^. However, most QKD protocols suffer from its relatively low rate of key generation, limiting its widespread applications in deployment. This is caused by the nature of quantum computing where it uses polarization to encode only one qubit for each photon. A costly remedy exists with little practical use–qutrit or ququart exploitation can be achieved by adding much more complications to the QKD apparatus.

This paper addresses the problem of efficient generation of cryptographic keys in QKD. This problem has been investigated by many researchers^3^,^4^,^5^. Zhou *et al*.^3^ present a four-intensity measurement-device-independent QKD protocol with a decoy state, which significantly improves the rate of key generation. This protocol works well if messages are not too long. Ma *et al*.^4^ argue that the rate of key generation for QKD can be increased by using an entanglement parametric down-conversion (PDC) source. Other methods aiming to improve the rate of key generation include information encoding using high-dimensional (HD) photonic degrees of freedom (such as position momentum^6^ and time energy^7^,^8^). At a practical level, however, it is difficult to increase the number of dimensions of the states encoded by phase over two, or at most, four.

Therefore, it is desirable to devise a more practical approach of encoding high dimensional states into a photon. On the one hand, spontaneous parametric down conversion (SPDC) is a simple way to produce squeezed and polarization-entangled light. On the other hand, recent advancements of orbital angular momentum (OAM) techniques are able to achieve faster generation of quantum states^9^, and to enable better control^10^ and easier integration with other systems^11^. First QKD protocols based on OAM have been proposed in refs 12, 13, 14. Based on these, Simon *et al*.^15^ propose Fibonacci-valued OAM states for high-capacity QKD protocols together with SPDC. Their protocols are easier and simpler to implement than existing SPDC and OAM protocols. However, the rate of key generation can be improved up to 4 times only, which is inadequate for any practical use. Also it is impossible to support long-distance transmission with lower error rate. This means that protocols trade transmission distances with error rates (the further the transmission distance the higher the error rate and vice versa).

It seems that achieving a considerable key rate with a small data size is rather challenging in practical settings. In this paper, we extend and enhance the use of the protocol of Simon *et al*.’s, by enabling each detected Fibonacci number to encode up to a decent number of secret key bits per transmitted entangled photon, while achieving transmission over longer distance with lower error rates. To be exact, we present an approach that uses matrices together with slightly modified QKD protocol^15^ to improve the rate of key generation. We observe that the conjugate relation (i.e., *L*_*n*+2_ = *F*_*n*+1_ + *F*_*n*−1_) between Lucas and Fibonacci numbers^16^, can be used to reduce side channel information leakage at the key preparation stage and hence to increase the security of QKD protocols^5^. Our observation is valid due to the contribution by Simon *et al*.^15^ who have shown that both Fibonacci-valued and Lucas-valued states can also be generated passively by using a beam splitter or by monitoring the idler of a SPDC source.

We propose a quantum cryptography protocol that is based on Fibonacci and Lucas matrix coding. Our new protocol effectively addresses the problem of random key generation for OTP. In particular, our proposed QKD protocol have the following characteristics.

1. It is a slight modification of the QKD protocol based on Simon *et al*.^15^. However, our protocol is free from the restrictions of orbital angular momentum and down-conversion bandwidths.
2. The key generation rate and the key update rate in our protocol are significantly higher than the existing solutions due to the use of the recurrence relations in Fibonacci or Lucas matrices.
3. A signal information leakage can be substantially reduced. This is because we use both Lucas-valued and Fibonacci-valued OAM entangled states, but the values carried by the transmitted entangled photons are all Fibonacci numbers. It is more difficult for the adversary Eve to know the signal information with entangled photons in a spontaneous parametric down conversion (SPDC) source^17^,^18^.
4. The verification of the integrity of encryption/decryption is possible due to the unique mathematical property of a Fibonacci or Lucas matrix. This feature does not exist in any previous QKD protocols.

## Results

We illustrate how to use the Fibonacci and Lucas matrix coding to develop a new high-capacity QKD protocol. We also provide a security analysis of the new protocol.

### Fibonacci and Lucas Matrix Coding

According to the definitions of Fibonacci and Lucas numbers (for details, see Appendixes I and II), we discuss how they can be used to construct relevant Fibonacci matrices 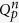 and Lucas matrices 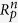. Then we explore their basic properties. Finally we investigate the relation between 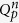 and 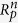.

The process of creating a sequence of 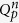 is iterative. We start from 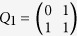 and then construct subsequent matrices *Q*_2_, *Q*_3_, …, *Q*_*p*_ according to the following relations:


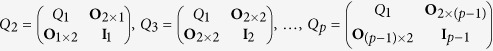


where **O**_*ij*_, *i* = *j* = 1, 2, …, *p* − 1 is a matrix of the dimension *i* × *j* with zero entries and **I**_*i*_, *i* = 1, 2, …, *p* − 1 is an identity matrix of the order *i*. It is easy to show that the matrices 
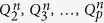
 satisfy the following relations:


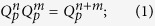



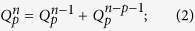



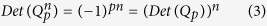


According to Eq. (3), 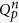 has its inverse, where *p* = 0, 1, 2, 3, …. As explained in Appendix I, it is easy to find its inverse 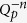 (see Eqs (26) and (27)). For example, the first four matrices 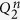 and their inverses are shownin Table 1.

The Lucas matrix (for details, see Appendix II) *R*_1_ can be used to generate matrices of higher dimensions *R*_2_, *R*_3_, …, *R*_*p*_ according to the following recurrence:


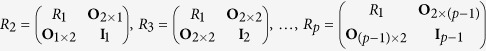


where **O**_*ij*_, *i* = *j* = 1, 2, …, *p* − 1 is a zero matrix of the dimension *i* × *j*, and **I**_*i*_, *i* = 1, 2, …, *p* − 1 is an identity matrix of order *i*. Matrices 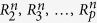 satisfy the following relations:


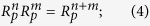



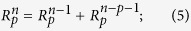



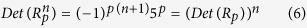


For 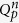, it is easy to find its inverse 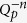 (see Eqs (33) and (34) given in Appendix II).

#### Fibonacci and Lucas matrix encryption and decryption algorithms

Let the plaintext be a sequence of integers: *p*_1_, *p*_2_, *p*_3_, *p*_4_, *p*_5_, *p*_6_, *p*_7_, *p*_8_, *p*_9_, …

These integers can be represented in the form of a square matrix *M* of order *p* + 1, *p* = 0, 1, 2, …. Note that the elements of *M* can be taken as an odd or even number of digits as we want. Therefore, the matrix encryption and decryption algorithms can be defined at a high level as follows^19^:


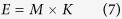


and


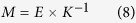


where *K* can be 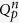 or 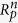, and *K*^−1^ is the inverse matrix of *K*.

### High-capacity quantum cryptographic protocol

**Figure 1:**
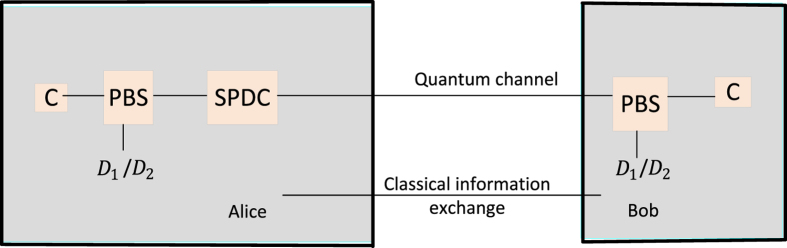
A schematic diagram for the Fibonacci- and Lucas- valued entanglement spontaneous parametric down conversion (SPDC) QKD. Alice and Bob connect to an entangled SPDC source by optical links. There is a *C*, a *D*_1_ and a *D*_2_ OAM sorter in Alice’s and Bob’s laboratories respectively. Either of the two entangled photons coming out from the SPDC source goes to Alice’s and Bob’s laboratories, and then the entangled photon randomly goes through the *C, D*_1_ or *D*_2_ sorter. The *C* sorter is used for allowing photons to arrive at the arrays of single-photon detectors when they are Fibonacci values. The *D*_1_/*D*_2_ sorter is used for filtering and blocking any non-Fibonacci values against various possible problems, and the *D*_1_ and the *D*_2_ sorters are used for allowing “diagonal” superpositions of the form 
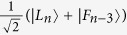
 and 
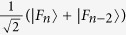
, respectively. Here, PBS stands for a polarized beam splitter.

**Figure 2:**
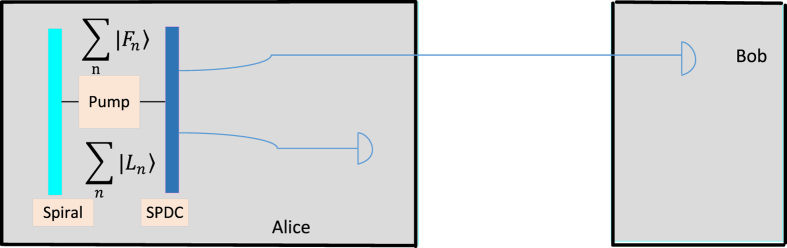
The experimental setup for the QKD protocol based on passive detected-state Fibonacci- and Lucas-valued entangled states. 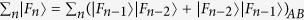
; 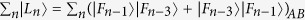. 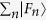 and 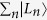 are two-photon output Fibonacci and Lucas entangled states, respectively.

To extend and enhance the framework of Simon *et al*.’s^15^, we use the Fibonacci and Lucas-valued OAM states detected in a transmission between Alice and Bob. The states are used to construct the Fibonacci matrix 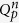 and the Lucas matrix 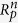 as the key (see Figs 1 and 2). Note that here the Fibonacci and Lucas values reconstructed from Fibonacci and Lucas-valued OAM states are used as the **seed** for generating recursive matrices 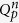 and 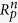 in terms of the assumption given below. We take an advantage of the recurrence relation of Fibonacci and Lucas matrices to significantly improve the information capacity of entangled photons to carry more than 4 bits of a cryptographic key.

#### Assumption

Assume that the order of the key matrix (the Fibonacci matrix 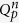 or the Lucas matrix 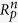) is determined by the quantum random number generators of Alice and Bob. The positions of Fibonacci and Lucas numbers in 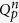 and 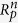 are determined by the outcome of *F*_*n*_ mod 4 or *L*_*n*_ mod 4, respectively. For instance, if *F*_*n*_ = 13, then *F*_*n*_ mod 4 = 13 mod 4 = 1. For *F*_*n*_ = 8, *F*_*n*_ mod 4 = 8 mod 4 = 0. Note that the positions of the Fibonacci numbers 13 and 8 in the matrices 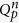 are illustrated below:


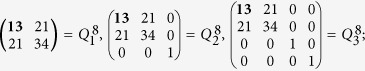



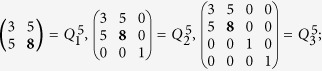


The steps of our protocol are described below.

**Step 1** **Preparation of Fibonacci and Lucas-valued OAM states.**

We slightly modify the experimental setup of Simon *et al*.’s^15^ (see Figs 1 and 2). Alice prepares Fibonacci and Lucas-valued OAM states and makes two-photon output states according to the following encoding


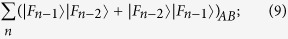



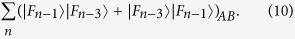


One photon of the entangled pair goes to the Alice laboratory and the other to the Bob laboratory. The selection of destination is random. Note that the main difference between our proposed protocol and Simon *et al*.’s^15^ is that we use the Fibonacci or Lucas values recovered from photons as a **seed** for constructing the Fibonacci matrix 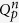 or the Lucas matrix 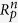. This trick improves the information capacity of photons considerably. We are free to select the **proper** pump values, say, 8, 11, 13, 18, 21, 29, 34, 47.

**Step 2** **Eavesdropping detection.**

As in the Simon *et al*.’s protocol^15^, there are six possible cases that need to be considered for entangled photons that arrive at Alice’s and Bob’s laboratories. They are listed below.

Case I. Both photons go to *C*.

Case II. One photon goes to *C* and the other to *D*_1_.

Case III. One photon goes to *C* and the other to *D*_2_.

Case IV. Both photons go to *D*_1_.

Case V. Both photons go to *D*_2_.

One photon goes to *D*_1_ and the other to *D*_2_.

In order to determine the case and detect eavesdropping, Alice and Bob need to exchange classical information over a classical channel (the channel can be an unprotected broadcasting). Let us introduce three events encoded as 0, 01 and 10. The event 0 occurs when the entangled photon goes to *C*. The second 01 when it goes to *D*_1_ and the third encoded as 10 when it goes to *D*_2_. Assume that there exists an eavesdropper Eve who intercepts an entangled photon, which travels to Alice (or Bob). Clearly, Eve has no information of which type of a detection measurement (*C, D*_1_ or *D*_2_) takes place in Bob’s laboratory. So, Eve has to guess. Eve makes a mistake if the photon goes to

- *C* in Eve’s laboratory while Bob’s laboratory applies either *D*_1_ or *D*_2_ or
- *D*_1_ in Eve’s laboratory while Bob’s laboratory applies either *C* or *D*_2_ or
- *D*_2_ in Eve’s laboratory while Bob’s laboratory applies either *C* or *D*_1_

The Alice measurement is going to be erroneous with the probability of 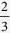. Eve’s activity is detected by Alice when Alice and Bob compare their transcripts.

**Step 3** **Reconstruction of the seed for the key matrix.**

After eavesdropping detection, if the error rate *r*_*e*_ is larger than the preset threshold *r*, Alice and Bob discard this communication and return to Steps 1–2. Otherwise, they can exchange classical bits to determine the correct Fibonacci number. Alice and Bob discard the trial if the exchanged classical bits (between Alice and Bob) satisfy one of the following three cases: (I) are both 01 (*D*_1_ sorters), (II) are both 10 (*D*_2_ sorters), (III) are 01 and 10 (*D*_1_ and *D*_2_ sorters), i.e., Cases IV-VI. If the exchanged classical messages are 0, 01 or 0, 10, Alice and Bob need to exchange one more classical message. That is, Alice or Bob need to exchange another classical bit 0 or 1, to let each other know that their trial is either Case II or Case III. This is sufficient for Alice (Bob) to know Bob’s (Alice’s) state.

If the exchanged classical bits between Alice and Bob are 0, they know the trial is Case I. They know each other’s value as the values can be identified from Eqs (9) and (10). In other words, Alice knows that the Bob Fibonacci number is one or two positions before her number (and vice versa, because the angular uncertainty principle links angular position and angular momentum 
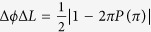
). However, this case is more complicated than Case II and Case III. To identify the correct OAM value, they need to exchange more classical messages. As we say in Step 1, the pump values of 8, 11, 13, 18, 21, 29, 34, 47 are used, so, the Fibonacci numbers encoded in the entangled photons can be 3, 5, 8, 13, 21, 34. As we can see the Fibonacci number is either even or odd, then, by prior agreement, for Alice, the classical bits 0, 1 are used to denote the first and second even Fibonacci numbers of 3, 5, 8, 13, 21, 34, while the classical bits 00, 01, 10, 11 are used to denote the first, second, third and fourth odd Fibonacci numbers of 3, 5, 8, 13, 21, 34 (see Table 2).

On receiving a classical bit from Alice, Bob can obtain the Alice Fibonacci number, because the number position is adjacent to the position of his Fibonacci number. Likewise, Bob sends the corresponding classical message to Alice in terms of Table 2, and Alice can also confirm the Bob Fibonacci number. That is to say that Alice and Bob are able to confirm to each other’s the detected numbers by exchanging classical information. For example, if Alice’s detected number is 3, then Alice sends the classical message 00 to Bob over the classical channel and Bob’s detected number is 8. So, Bob can obtain the **seed** 11, and Bob sends the classical message 00 to Alice over the classical channel. Alice can also obtain the **seed** 11.

**Step 4** **Cryptographic key generation with** 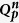 **and** 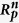


After the pump values of Fibonacci and Lucas numbers are determined by Alice and Bob, they can use them as the **seed** for the key matrix, i.e., the Fibonacci matrix 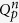 or the Lucas matrix 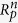. The orders of 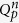 and 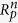 are determined by their quantum random number generators in their laboratories. If the obtained Fibonacci number is *F*_4_ = 5 and its matching order is 4, then the inverse of the matrix can be found in Table 1. The matrices can be used to perform basic cryptographic operations such as encryption and decryption. To illustrate them, consider the following example. Assume that a message is a sequence of integers


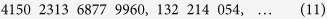


Integers of the message in Eq. (11) can be put as entries of matrices *M*_1_, *M*_2_, …, which is


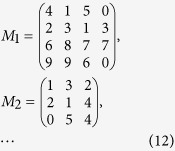


According to Eq. (7), *M* can be encrypted as follows


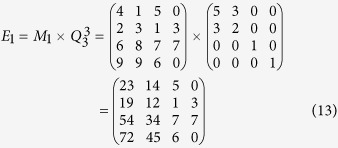



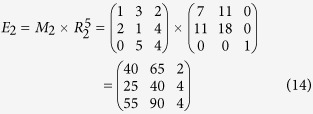


According to his order and Equation (8), the cryptogram 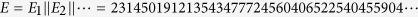 can be split and decrypted by using the inverse matrix, so Bob obtains


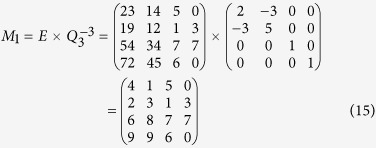



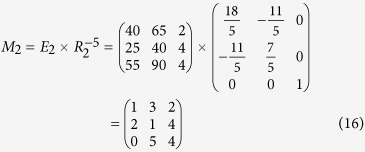



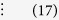


**Step 5** **Integrity verification**

According to 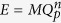, 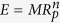, we obtain


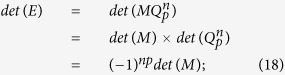



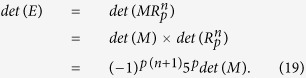


If the transmitted and received matrices are *E* and *E*′ respectively, then *E*′ satisfies Eqs (18) or (19). Hence, the integrity of *E* is proved.

### Security Analysis

The encryption defined by Fibonacci and Lucas matrices is an instance of symmetric-key cryptography. Fibonacci and Lucas matrices have the particular property, i.e., the recurrent property which helps us to know a Fibonacci or Lucas matrix with any one of Fibonacci or Lucas number in the matrix. We call the a Fibonacci or Lucas number the **seed**. As we know, for symmetrical cryptography, the main deficiency is the problem of key distribution. However, in our proposed protocol, the key is generated based on Simon *et al*.’s protocol^15^, which is quantum-resistant for enhanced security. That is, Fibonacci or Lucas cryptography can be combined with the quantum one-time-pad for unconditional security. Therefore, in this section, we provide a sufficient security analysis of why seeding the Fibonacci and Lucas matrices used to encrypt the message (and subsequent sending of it) does not increase Eve’s information about the secret key as follows.

1. Firstly, similar entangled states are prepared with the improved experimental setup, by the virtue of the recurrence relations of *F*_*n*+2_ = *F*_*n*+1_ + *F*_*n*_ and *L*_*n*+2_ = *F*_*n*+1_ + *F*_*n*−1_. Clearly, the entangled photons detected by Alice, Bob and the adversary Eve are Fibonacci-valued. When Eve receives the entangled photon and even she can detect its values, it is more difficult for her to distinguish Fibonacci entangled states from Lucas entangled states than that in Simon *et al*.’s protocol^15^. More precisely, if Eve makes a *D*_1_-type measurement on an entangled photon heading to Bob, which is actually in the eigenstate |*F*_*i*_〉. Then she will detect one of the two superposition states |*F*_*i*−2_〉 + |*F*_*i*_〉 or |*F*_*i*_〉 + |*F*_*i*+2_〉, with the probability of 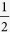, respectively. Then Eve transmits either of these two superposition states to Bob. If Bob receives one of these superpositions and makes a *C*-type measurement, he will read out one of the values *F*_*i*_, *F*_*i*−2_, or *F*_*i*+2_, with the respective probabilities of 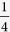, 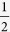, 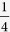. However, he should obtain |*F*_*i*_〉 with the probability of 1 if there is no eavesdropper. If Eve makes a *D*_2_-type measurement on an entangled photon heading to Bob, which is actually in the eigenstate |*F*_*i*_〉, then she will detect one of the two superposition states |*L*_*i*−2_〉 + |*F*_*i*_〉 or |*F*_*i*_〉 + |*L*_*i*+2_〉, with the probability of 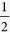, respectively. Then Eve transmits either of these two superposition states to Bob. If Bob receives one of these superpositions and makes a *C*-type measurement, he will read out one of the values *F*_*i*_, *L*_*i*−2_, or *L*_*i*+2_, with the respective probabilities of 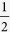, 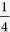, 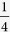. However, he should obtain |*F*_*i*_〉 with the probability of 1 if there is no eavesdropper. In these situations, these superposition states do not help Eve to know the **seed**, but expose her eavesdropping action. Consequently, Alice and Bob discard such entangled photons.
2. Secondly, if Eve makes a *C*-type measurement on an entangled photon heading to Bob, which is actually in the eigenstate |*F*_*i*_〉, then she will detect |*F*_*i*_〉 with the probability of 1. Then Eve transmits |*F*_*i*_〉 to Bob. If Bob receives it and makes a *C*-type measurement, he will also read out one of the values *F*_*i*_, with the probability of 1. Though Eve’s eavesdropping action cannot be detected, it is still impossible for her to know the definite OAM value. This is because in Step 3, which classical bit representation of which Fibonacci number is agreed with between Alice and Bob in advance, and the classical channel is the broadcast channel. In other words, when Alice/Bob sends classical messages to Bob/Alice through the classical channel, Bob/Alice can determine the **seed**, but Eve cannot without knowing Alice and Bob’s prior agreement, she just knows 0 and 1 that are used to denote even Fibonacci numbers, and 00, 01, 10, 11 to denote odd Fibonacci numbers. So, for Eve, it is at random. Eve has no choice but to guess the obtained Fibonacci and Lucas numbers by Alice and Bob with a probability of 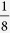. Moreover, to prevent Eve from capture two small pulses for the two entangled photons at the same time, the time interval for sending entangled photons is random.
3. Thirdly, the positions of obtained Fibonacci and Lucas numbers in matrices 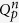 and 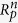 are not fixed. Instead, the positions are determined by *F*_*n*_/*L*_*n*_ mod 4. It suggests that our method can be further against Eve’s attack. If Eve guesses the wrong obtained Fibonacci or Lucas number by Alice and Bob, she places the position of the wrong obtained Fibonacci or Lucas number in 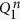 and 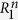 with a probability of 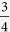.
4. Lastly, the construction of the final key matrices including the Fibonacci and Lucas matrices is determined by these matrices’ orders *p*, which are determined by the quantum random number generators in Alice’s and Bob’s laboratories. Therefore, Eve cannot know the value *p*. Furthermore, the order determines how to split the encrypted message when decrypting them. Even if Eve guesses the correct seed, it is very difficult for her to guess all the right orders for all seeds during the construction of the final key matrices. As long as Eve does not guess all the orders for all corresponding matrices, she also splits the encrypted message in a wrong way. Moreover, in our protocol, we use matrix multiplication to encrypt the message. In matrix multiplication, if the orders of two square matrices are not equal, they cannot be performed multiplication operation. In other words, Eve cannot know any information about the messages if she guess a wrong order matching the matrix. Most importantly, there are no relations among these established Fibonacci or Lucas matrices. So, for Eve, *p* is at random, the probability for Eve to know the right 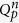 or 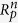 is 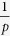, and the probability for Eve to know a right 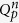 or 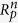 is 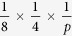, i.e., 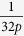.

Therefore, by seeding the Fibonacci and Lucas matrices used to encrypt the message to achieve fast and simple high-capacity quantum cryptography, our proposed protocol does not increase Eve’s information about the secret key.

## Discussion

In this section, we discuss the possibility to improve Simon *et al*.’s^15^ experimental setup for quantum cryptography based on the Fibonacci matrix 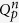 and the Lucas matrix 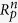, which have more additional features to be obtained, including the higher transmission rates, no limit of communication bandwidths, the considerable information capacity, the selection property of Fibonacci or Lucas numbers, and the powerful detection and correction ability.

### Entangled Fibonacci- and Lucas- sequence spiral source

In 2013, Simon *et al*.^15^ proposed a high-capacity QKD by Fibonacci coding. In particular, with a Vogel spiral^15^ (which refers to the “Fibonacci angle”), they use a source of entangled Fibonacci-valued OAM states to prepare Fibonacci-valued entangled pairs, which then leave the spiral and enter the down-conversion crystal. Moreover, due to the conjugation relation between Lucas numbers and Fibonacci numbers, Simon *et al*.^15^ showed that Lucas-valued states can also be generated passively by using a beam splitter or by monitoring the idler of an SPDC source. In addition, the phases of signal photons are totally random due to the spontaneous feature of the SPDC process. This intrinsic phase randomization improves the security of the QKD system by making it immune to source attacks. Therefore, we improve the experimental setup in Simon *et al*.’s protocol to generate both Fibonacci- and Lucas-valued OAM states (see Figs 1 and 2, note that Simon *et al*.^15^ state that in the chosen operating range, it is easy to adjust the fraction of the values that fall on the Fibonacci or Lucas sequence), so that these nonorthogonal states naturally appear and randomly change with Fibonacci- and Lucas-valued entangled pairs.

### The overall transmission rate

Assume that the detection probabilities of the entangled photons in the state of Eqs (9) and (10) are independent. Let *ξ*_*A*_, 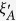 and *ξ*_*B*_, 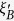 be the detection efficiencies for a Fibonacci and Lucas entangled photon for Alice and Bob, respectively. Both *ξ*_*A*_, 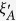 and *ξ*_*B*_, 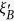 take into account the channel losses, detector efficiencies, coupling efficiencies, and losses inside the detector box. For a 2*n*-photon pair, the overall transmission rate is


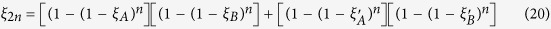


Given that the channel loss is included in *ξ*_*A*_, 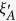 and *ξ*_*B*_, 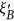, our method can be used to the SPDC source on either Alice’s (or Bob’s) side or between Alice and Bob.

### The information capacity

Due to the recursive property of the Fibonacci matrix 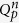 and the Lucas matrix 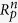, just one entangled photon can be used as the seed to distribute the entire key, i.e., the information capacity *I*_*c*_ can be even equal to the length of the key 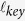, i.e., 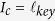. This is because the secret can be used to construct a matrix of any order.

### No limit of communication bandwidths

Simon *et al*.’s^15^ protocol needs more Fibonacci or Lucas numbers to improve key capacity, however, the method is under the limit of orbital angular momentum and down-conversion bandwidths. Therefore, they cannot choose more proper Fibonacci or Lucas numbers to achieve longer distances and lower error rates simultaneously. Moreover, every entangled photon can only be used to encode at most four bits. So, a large number of entangled states should be prepared to establish the key. As a result, when the key is updated frequently with the purpose of security, one-time-pad communication bandwidth increases in a proportional way. However, our protocol improves the key capacity greatly by taking advantages of the recursive property of Fibonacci and Lucas matrices and the method of preparing entangled states by Simon *et al*.’s^15^ setup. Therefore, only a few entangled photons are needed to establish the key in practical settings. Therefore, our protocol is free from the limitation of orbital angular momentum and down-conversion bandwidths. Given this, in our protocol, the key can be established in a short time, and the frequent key update is free from the limitation of communication bandwidths.

### The selection property of Fibonacci or Lucas numbers

Simon *et al*. used their experiments to come to the conclusion that if the pump values from smaller Fibonacci numbers are used, the photons can travel longer distances but at the expense of higher error rates. If, however, the pump values from larger Fibonacci numbers are used, then error rates reduced but maximal distances over which photons can travel are shorter. That is, to meet the requirements of available orbital angular momentum and down-conversion bandwidths and the longer distances and lower error rates, the **proper** pump values can be selected in our protocol, for example, Fibonacci numbers 8, 13, 21, 34 and Lucas numbers 11, 18, 29, 47. Therefore, the values carried by entangled photons are 3, 5, 8, 13, 21, 34.

### Error detection and correction abilities

An additional feature of our protocol is the error detection for the cipertext compared with the existing quantum cryptography, which can keep the integrity of the secret. Stakhov^19^ has proposed an error correction algorithm for Fibonacci coding. We present a brief description of this algorithm. For 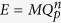, 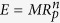, we can verify their integrity in terms of Eqs (15) or (16), i.e., 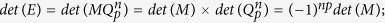 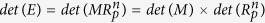 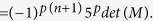


If the transmitted matrix is *E* and the received matrix is *E*′, and *E*′ satisfies Eqs (15) or (16), then there are no errors. Otherwise, there exist errors. In this case, correction is needed, and Stakhov^19^ has shown that the correction ability of Fibonacci 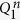 and 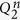 matrix coding method is 93.33% and 99.80%, and when *p* is larger, the correction ability is higher than 99.80%, which exceeds all the other well-known correcting codes.

Because of the considerable information capacity, no limit of communication bandwidths, and the powerful detection and correction abilities, our protocol provides a practical secure way to share more private information with high photon-information efficiency in a short time. In realistic conditions, it is more applicable and feasible in a practical implementation with a slight modification of Simon *et al*.’s protocol. Table 3 compares the features of our proposed protocol with those of the most relevant quantum key distribution protocols in refs 1, 2 and 15. The comparison suggests that our protocol is more suitable for real-world applications.

## Conclusions

We have developed a new quantum cryptosystem, i.e., quantum cryptography based on Fibonacci matrix 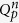 and Lucas matrix 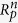, which employs technologies similar to Simon *et al*.’s protocol to overcome the previous limitations on communication bandwidths and demonstrate that the number of secret key bits per transmitted entangled photon can be increased up to the length of the key, which is well-over previous demonstrations that were limited to up to 4. Under realistic conditions, the proposed protocol also provides a practical secure way to share more private information with considerably high photon-information efficiency in a short time. Moreover, it can be fast and simple to implement technical realization.

## Methods

Here, we introduce the method of Fibonacci matrix 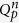 or Lucas matrix 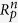 to quantum cryptography.

Let the initial message be a “digital signal” of integers: *p*_1_, *p*_2_, *p*_3_, *p*_4_, *p*_5_, *p*_6_, *p*_7_, *p*_8_, *p*_9_, …

Assume that we choose the first nine readings and form a 3 × 3 matrix *P*_1_, which is regarded as a plain text matrix.


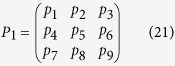


And the key *K*, i.e., 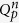 or 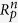 is obtained by Steps 1–3 in the proposed protocol. In general, the cryptosystem consists of the plain text matrix *P*, the cipher text matrix *C*, and the key *K*


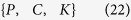


Then encryption and decryption algorithm is

If 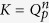


then 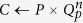;


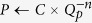


Endif

If 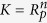


then 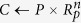;


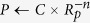


Endif

**Appendix I**

**Definition 1.** (**Fibonacci numbers**)^20^ The sequence 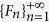 of Fibonacci numbers is defined by the recurrence relation


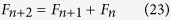


Clearly, the integers 1, 2, 3, 5, 8, 13, 21, 34, 55, 89, … are members of Fibonacci sequences. Using the definition of Fibonacci numbers, one can prove that


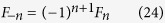


Now we are ready to introduce a Fibonacci *Q*-matrix^21^,^22^ as


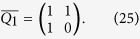


According to Stakhov’s work^19^, we can write a Fibonacci *Q*-matrix of dimension 2 as follows:


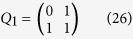


Now, we can derive a relevant recurrence relation in the form:


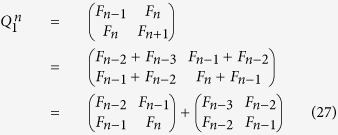



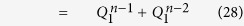


Note that 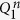 satisfies the following two properties:

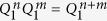
 and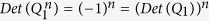.

The inverse matrix 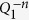 of 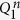 is obtained as follows


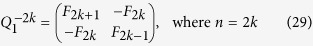



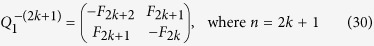


For example, according to Eqs (26) and (27), we can obtain 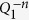 of 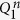 when *n* = 4, 5, 6, 7 (see Table 4).

**Appendix II**

**Definition 2.** (**Lucas numbers**)^16^ The Lucas numbers are defined as follows:


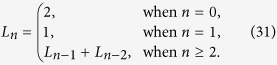


In particular, *L*_0_ = 2, *L*_1_ = 1, *L*_2_ = 3, *L*_3_ = 4, *L*_4_ = 7 … Moreover, Lucas numbers and Fibonacci numbers have a conjugate relation^16^ of the following form:


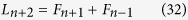


Let us define a 2 × 2 matrix *R* as


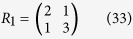


Therefore, according to Eqs (20), (29) and (30), we have


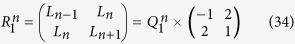



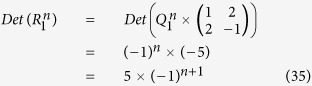


The inverse matrix 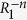 of 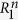 can also be derived in terms of Eqs (26), (27), (31) and (32), which is as follows


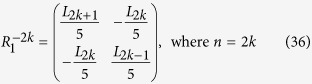



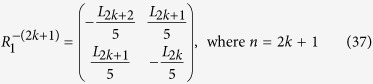


## Additional Information

**How to cite this article:** Lai, H. *et al*. Fast and simple high-capacity quantum cryptography with error detection. *Sci. Rep.* **7**, 46302; doi: 10.1038/srep46302 (2017).

**Publisher's note:** Springer Nature remains neutral with regard to jurisdictional claims in published maps and institutional affiliations.

## Figures and Tables

**Table 1:** 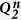
 and 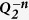, where *n* = 2, 3, 4, 5.

*n*	2	3	4	5
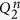	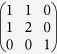	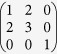	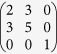	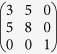
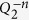	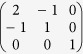	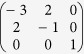	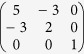	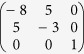

**Table 2:** The possible Fibonacci values obtained by Alice and their corresponding classical representations.

The possible Fibonacci values obtained by Alice	3	5	8	13	21	34
The classical bits sent by Alice/Bob	00	01	0	10	11	1

**Table 3:** Performance comparison of our QKD with the most relevant previous QKDs.

Protocols	Ref. 1	Ref. 2	Ref. 15	Our protocol
The maximal information capacity	1	2	4	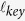
The correction ability	n/a	n/a	n/a	Higher than 93.33%
The ability to verify the integrity of ciphertext	No	No	No	Yes
The limitation to bandwidths	Yes	Yes	Yes	No
Achieving longer distances and lower error rates	No	No	No	Yes

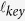
 denotes the length of the key, “n/a” which means not applicable.

**Table 4:** 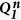
 and 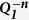, where *n* = 4, 5, 6, 7.

*n*	4	5	6	7
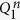	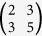	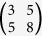	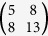	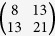
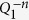	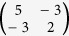	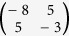	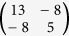	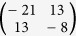
